# Histidine 19 Residue Is Essential for Cell Internalization of Antifungal Peptide SmAP_α1-21_ Derived from the α-Core of the *Silybum marianum* Defensin DefSm2-D in *Fusarium graminearum*

**DOI:** 10.3390/antibiotics11111501

**Published:** 2022-10-28

**Authors:** Agustina Fernández, Mariano González, Ismael Malbrán, Romina F. Vázquez, Sabina M. Maté, Fanny Guzmán, Laura S. Bakás, Sandra Vairo Cavalli

**Affiliations:** 1 CIPROVE-Centro Asociado CIC, Departamento de Ciencias Biológicas, Facultad de Ciencias Exactas, Universidad Nacional de La Plata, La Plata 1900, Argentina; 2 Centro Científico Tecnológico del Consejo Nacional de Investigaciones Científicas (CONICET, CCT-La Plata), La Plata 1900, Argentina; 3 Centro de Investigaciones de Fitopatología (CIDEFI-UNLP-CIC), Facultad de Ciencias Agrarias y Forestales, Universidad Nacional de La Plata, La Plata 1900, Argentina; 4 Instituto de Investigaciones Bioquímicas de La Plata “Prof. Dr. Rodolfo R. Brenner” (INIBIOLP—CONICET), Facultad de Ciencias Médicas, Universidad Nacional de La Plata, La Plata 1900, Argentina; 5 Núcleo de Biotecnología Curauma (NBC), Pontificia Universidad Católica de Valparaíso, Valparaíso 2373223, Chile

**Keywords:** defensins, antimicrobial peptides, antifungal peptides, *Fusarium graminearum*, antifungal peptide design, Fusarium head blight

## Abstract

The synthetic peptide SmAP_α1-21_ (KLCEKPSKTWFGNCGNPRHCG) derived from DefSm2-D defensin α-core is active at micromolar concentrations against the phytopathogenic fungus *Fusarium graminearum* and has a multistep mechanism of action that includes alteration of the fungal cell wall and membrane permeabilization. Here, we continued the study of this peptide’s mode of action and explored the correlation between the biological activity and its primary structure. Transmission electron microscopy was used to study the ultrastructural effects of SmAP_α1-21_ in conidial cells. New peptides were designed by modifying the parent peptide SmAP_α1-21_ (SmAPH19R and SmAPH19A, where His19 was replaced by Arg or Ala, respectively) and synthesized by the Fmoc solid phase method. Antifungal activity was determined against *F. graminearum*. Membrane permeability and subcellular localization in conidia were studied by confocal laser scanning microscopy (CLSM). Reactive oxygen species (ROS) production was assessed by fluorescence spectroscopy and CLSM. SmAP_α1-21_ induced peroxisome biogenesis and oxidative stress through ROS production in *F. graminearum* and was internalized into the conidial cells’ cytoplasm. SmAPH19R and SmAPH19A were active against *F. graminearum* with minimal inhibitory concentrations (MICs) of 38 and 100 µM for SmAPH19R and SmAPH19A, respectively. The replacement of His19 by Ala produced a decrease in the net charge with a significant increase in the MIC, thus evidencing the importance of the positive charge in position 19 of the antifungal peptide. Like SmAP_α1-21_, SmAP2H19A and SmAP2H19R produced the permeabilization of the conidia membrane and induced oxidative stress through ROS production. However, SmAPH19R and SmAPH19A were localized in the conidia cell wall. The replacement of His19 by Ala turned all the processes slower. The extracellular localization of peptides SmAPH19R and SmAPH19A highlights the role of the His19 residue in the internalization.

## 1. Introduction

The massive use of antifungal agents in agriculture has led to the appearance of resistant strains in plant pathogens, greatly restricting the number of compounds available [1] and probably contributing to the development of resistance against other antifungal compounds [2,3]. For Fusarium head blight (FHB), several factors further hamper the effectiveness of fungicide treatments, including lack of impact of available molecules, uneven flowering of wheat, and low retention of fungicides in the spikes [4]. The economic impact of FHB exceeds the decrease in crop yield and includes contamination of the grain with trichothecenes, a group of mycotoxins that hamper their use as food or feed [5].

Antimicrobial peptides (AMPs) are a group of host defense peptides and small, structurally diverse proteins that belong to the nonspecific innate immune system and act as part of the first line of immune defense [6]. AMPs are typically short cationic amphiphilic peptides present in bacteria, fungi, plants, and animals [7]. Plant AMPs act against a wide range of pathogens, exhibiting different levels of efficacy. This could be attributed to the fact that AMPs have taken advantage of the biochemical divergence and evolution of cell walls and membranes, which are the first AMP pathogen targets. At the same time, they turn out to be harmless to the host [8]. In recent years, AMPs have acquired considerable scientific interest due to their potential practical applications in engineering disease resistance in plants and as promising templates for the development of eco-friendly plant disease control agents and next-generation pharmaceuticals [9,10]. In plants, they accumulate in the peripheral cell layer, preferentially of nutrient-rich structures (i.e., flowers and seeds), where they are constitutively produced or induced by various defense or stress-signaling pathways [11]. A particular plant AMP family is the defensin family, which exhibits a cysteine-stabilized α/β tridimensional structural pattern, the so-called CSα/β fold, with low primary sequence similarity and broad-spectrum activity. 

Most variations between primary sequences occur in the loops of the plant defensins, especially in the region comprising the γ-core. The γ-core GXC(X_3–9_)C is a well-conserved motif that occurs not only in plant defensins but also in all cysteine-rich AMPs. Because of amino acid diversity in the loops, the three-dimensional position, and the variable length of the intercysteine segments, defensins have functional flexibility despite their small overall size and rigid architecture. Additionally, many of the amino acids that compose those loops are exposed in the molecule surface, granting the ability to interact with other proteins (for oligomerization) or molecule targets to exert the antimicrobial activity [12,13,14]. Many efforts have been made to identify minimal active motifs in defensins that would allow the design of new antimicrobial agents [12,15,16]. In some AMPs, the γ-core alone is sufficient for antimicrobial activity. This motif, however, also seems to be a scaffold to which complementary antimicrobial determinants are attached as modules in various configurations [17].

In addition to the γ-core, plant defensins also contain the α-core motif with the conserved sequence GXC(X_3–5_)C, which is located in the proximity of the N-terminal region. The α-core motif is a loop connecting the β1 strand with the α-helix; this motif is not conserved in all disulfide-containing AMPs [18]. The role of the α-core in antifungal activity has not been widely studied. While the defensins MsDef1 (*Medicago sativa*) and MtDef4 (*M. truncatula*) inhibit the growth of *Fusarium graminearum*, the chemically synthesized α-core motifs from both defensins (GPCFSGC and GPCASDHNC, respectively) are inactive against the same filamentous fungus, indicating that neither of the α-core motifs exhibits antifungal activity *per se* [18]. Peptides derived from the α-core region from a *Brassica hybrid* cv. Pule defensin exhibited activity against *Colletotrichum gloeosporioides*, albeit at millimolar concentrations [19].

Natural AMPs are a rich source for the rational design of novel AMPs with improved properties. An in-depth understanding of the structure and mode of action of AMPs is required for their potential future modification or application as antifungal compounds [20]. In a previous work [21], we showed that the α-core motif may serve as a promising scaffold for that purpose. DefSm2 is a putative antifungal protein with a defensin domain naturally expressed in flowers of the wild thistle *Silybum marianum*. The homology-based structural model of the defensin domain DefSm2-D predicts the α-core motif GNCGNPRHC. Chemically synthesized peptides including this α-core (SmAP_α1-21_ and SmAP_α10-21_) inhibit the growth of the phytopathogen *F. graminearum* in vitro at low micromolar concentrations. SmAP_α1-21_ exerts a remarkable effect on the cell wall’s outermost layer and induces membrane permeabilization and vesiculation in conidial cells [21]. 

In the present work, we deepened the characterization of SmAP_α1-21_ mode of action. We studied the ultrastructural effects of SmAP_α1-21_ in conidial cells by transmission electron microscopy (TEM). We explored the correlation between its biological activity and primary structure, focusing on the relevance of a His residue. For that purpose, we designed and synthesized two peptides based on SmAP_α1-21_ (SmAPH19R and SmAPH19A, where His19 was replaced by Arg or Ala, respectively). We characterized the biophysical interaction between the peptides and Langmuir monolayers representative of fungal membranes and determined the peptides’ antifungal activity against *F. graminearum*. Membrane permeability and reactive oxygen species (ROS) production were also studied. Additionally, we demonstrated here the significance of His19 in the internalization of peptide SmAP_α1-21_ as part of its mechanism of action.

## 2. Results

### 2.1. Antifungal Peptide Design, Synthesis, and Characterization

To deepen the study of the α-core motif—which is restricted to the plant defensin family and has not been extensively studied—and the mechanism of action of SmAP_α1-21_, in this work, we designed new peptides. Two peptides were synthesized based on SmAP_α1-21_ through the F-moc strategy, called SmAP2H19R and SmAP2H19A. Table 1 summarizes the main characteristics of the designed and synthesized peptides. 

### 2.2. Peptide SmAP2H19A Derived from SmAP_α1-21_ Is Less Active than Parent Peptide

**Figure 1:**
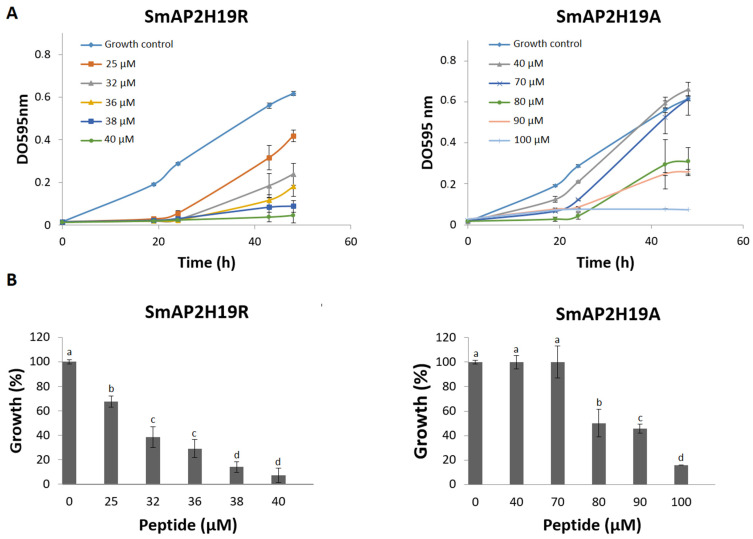
(**A**) Growth curves of *Fusarium graminearum* in the presence of different concentrations of SmAP_α1-21_-derived peptides. Error bars represent standard deviation of technical triplicates. DO 595 nm is optical density at 595 nm. (**B**) Percentage of growth of *F. graminearum* at final time point in panel A (48 h). Bars represent mean ± standard deviation of percentage of growth as compared with 100% from control growth, defined as fungus growth in absence of peptide. Treatments with same letter do not significantly differ (*p* > 0.05).

**Figure 2:**
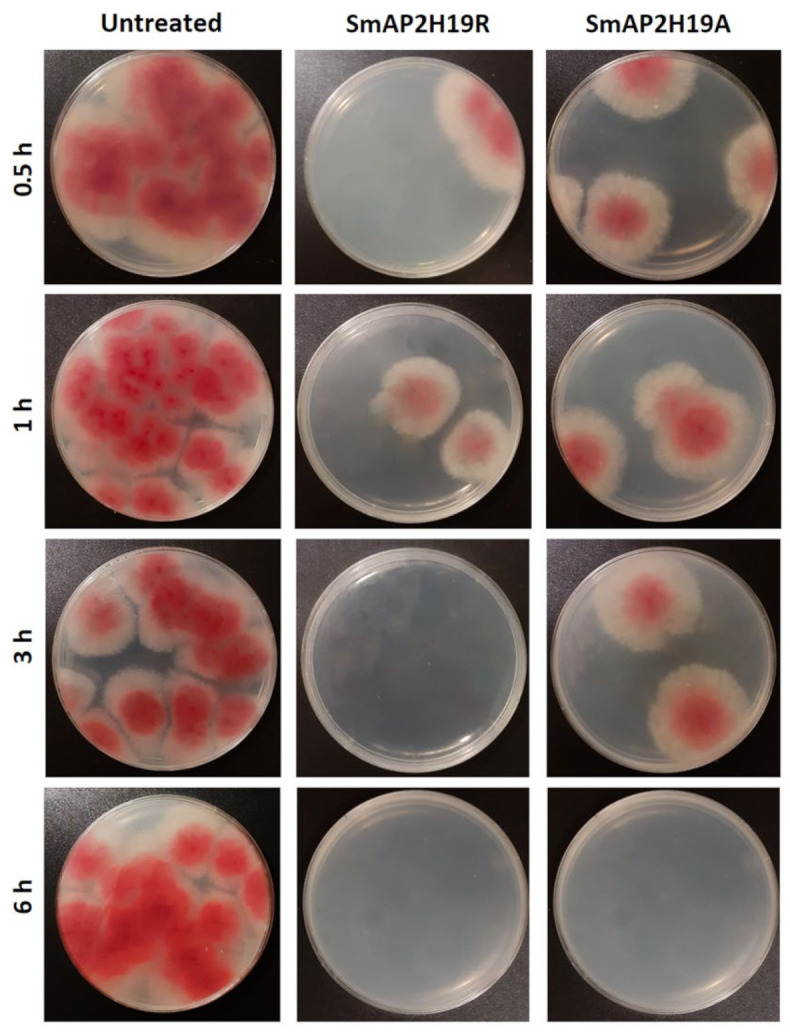
*Fusarium graminearum* growth in potato dextrose agar (PDA) plates incubated for 72 h at 25 °C in the presence of peptides SmAP2H19R and SmAP2H19A as it was described for time-to-kill experiment (*cf.* Material and Methods section). Four time treatments are shown: 0.5, 1, 3, and 6 h.

The antifungal properties of each peptide against *F. graminearum* conidia were examined using the MIC and time-to-kill assays (Figure 1 and Figure 2, respectively). The strongest growth inhibition was detected for SmAP2H19R. This peptide produced a significant growth inhibition at concentrations above 25 µM. At 38 µM, SmAP2H19R completely inhibited the germination of conidia. Below this concentration, it produced a 20 h delay in the germination of conidia at all the concentrations assayed (Figure 1A). The peptide SmAP2H19A exhibited a MIC of 100 μM (Figure 1B), whereas no significant growth inhibition was observed below 70 μM. 

Both SmAP2H19R and SmAP2H19A were lethal for conidia in the time-to-kill experiment (Figure 2). At the assay conditions (MIC), these peptides were able to exert their activity after 1–3 and 3–6 h of incubation, respectively. A small number of viable conidia, however, were observed after 0.5 h of incubation for both peptides at their MIC.

### 2.3. Peptides Are Inserted onto Lipid Monolayers and Permeabilize Fungal Membrane

Lipid monolayers composed of 1-palmitoyl-2-oleoyl-sn-glycero-3-phosphocholine (POPC)–ergosterol in a molar ratio of 3:1 were used as simple models of a fungal plasma membrane to test the insertion ability of the peptides.

**Figure 3:**
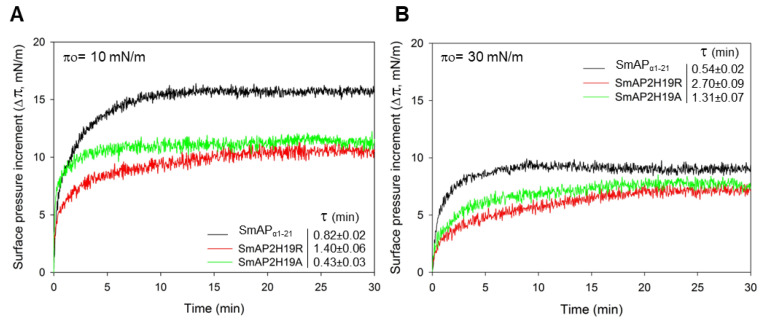
Peptide interaction with fungal-like lipid monolayers. Kinetics of insertion of SmAP_α1-21_ (black trace), SmAP2H19R (red trace), and SmAP2H19A (green trace) into POPC–ergosterol (3:1 mole ratio) monolayers at initial surface pressures (π_o_) of (**A**) 10 mN/m and (**B**) 30 mN/m. Peptides were injected beneath monolayers at the depicted π_o_ to give final concentration of 20 μM in subphase bulk, and the increase in surface pressure (Δπ) was monitored over time. Time (τ) needed to achieve half of Δπ_eq_ is presented for each peptide at both π_o_ values and was obtained by fitting Δπ vs. time curves to Equation (1) (*cf.* Material and Methods section). Measurements were performed at 23 ± 1 °C.

**Figure 4:**
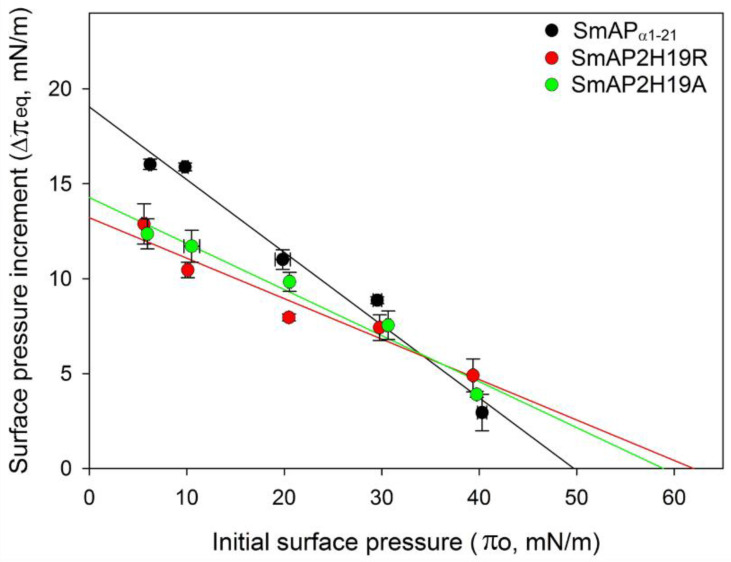
Increment in surface pressure at equilibrium (Δπeq) obtained after injection of peptides (20 μM) beneath the POPC–ergosterol monolayers at different initial surface pressures (π_o_) of lipid films. Maximum insertion pressure (MIP parameter) for each peptide was obtained from these plots by extrapolating linear regression curves to Δπ_eq_ = 0.

The increase in surface pressure (Δπ) due to peptide interaction with the lipid monolayer was measured at several initial surface pressures (π_o_) of the lipid films by the Langmuir balance method. Figure 3 shows representative surface pressure curves as a function of time for SmAP_α1-21_, SmAP2H19R, and SmAP2H19A at π_o_ values of 10 (A) and 30 mN/m (B). After injection of the peptides in the subphase beneath the lipid films, a rapid increase in the surface pressure was observed in each case, which accounts for the fast association kinetics of the peptides to the POPC–ergosterol monolayers. The overall kinetics showed half-time values to reach the equilibrium state (τ) between 0.4 and 3 min, with SmAP2H19R presenting slightly slower kinetics than their counterparts (τ values for the insertion at 10 and 30 mN/m are shown as insets in Figure 4A,B, respectively). With increasing lipid packing densities—i.e., higher π_o_—lower Δπ_eq_ were registered for either peptide since the incorporation into lipid monolayers is commonly prevented at high lipid packing [22]. This feature was more pronounced in the case of the His-containing peptide, which produced higher increments in surface pressure at low π_o_ of the lipid monolayers than the Arg- or Ala-substituted derivatives, as shown for monolayers at π_o_ = 10 mN/m (Figure 3A). Notwithstanding, the three peptides showed similar effects in terms of Δπeq at higher π_o_, as is the case for π_o_ = 30 mN/m (Figure 3B), a surface pressure considered representative of the lipid packing in a cell membrane [23]. The plots of Δπ produced at equilibrium upon peptide injection for different π_o_ of the lipid films are shown in Figure 4. From these curves, the maximum insertion pressure (MIP), a suitable parameter for the characterization of peptide–lipid interaction, was obtained for each peptide by extrapolating the corresponding linear regression curves to Δπ = zero. The MIP parameter represents the maximum surface pressure beyond which no peptide insertion would occur and is correlated with the peptide’s affinity for the lipid monolayer [24]. Calculations revealed MIP values of 49.7 ± 3.4, 62.0 ± 8.8, and 58.8 ± 6.2 mN/m for SmAP_α1-21_, SmAP2H19R, and SmAP2H19A, respectively. 

**Figure 5:**
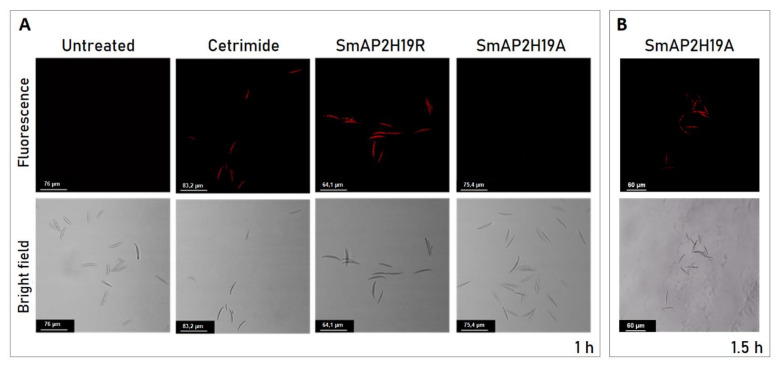
(**A**) Bright field (bottom) and fluorescence (above) images of *Fusarium graminearum* conidia incubated with peptides SmAP2H19R and SmAP2H19A at their MIC for 1 h. Cetrimide was used as positive control. (**B**) Bright field (bottom) and fluorescence (above) images of *F. graminearum* conidia incubated with SmAP2H19A at its MIC for 1.5 h. Bar indicates size in μm.

Membrane integrity was evaluated in *F. graminearum* conidia treated with the peptides SmAP2H19R and SmAP2H19A using the PI probe through CLSM. PI is a membrane-impermeant fluorogenic nucleic acid dye that stains necrotic and late apoptotic cells with damaged plasma membranes. PI was observed in conidia treated with the peptide SmAP2H19R for 1 h, as evidenced by the presence of intense red fluorescence. On the contrary, conidia exposed to the peptide SmAP2H19A were impermeant to PI and needed a longer incubation time (1.5 h) for the membranes to become permeant to the dye (Figure 5B). SmAP_α1-21_ induced conidia aggregation as was previously reported [21]; however, this effect was not observed with any of the modified peptides tested in this work. 

### 2.4. SmAP_α1-21_ Induces Endogenous ROS and Peroxisome Biogenesis

**Figure 6:**
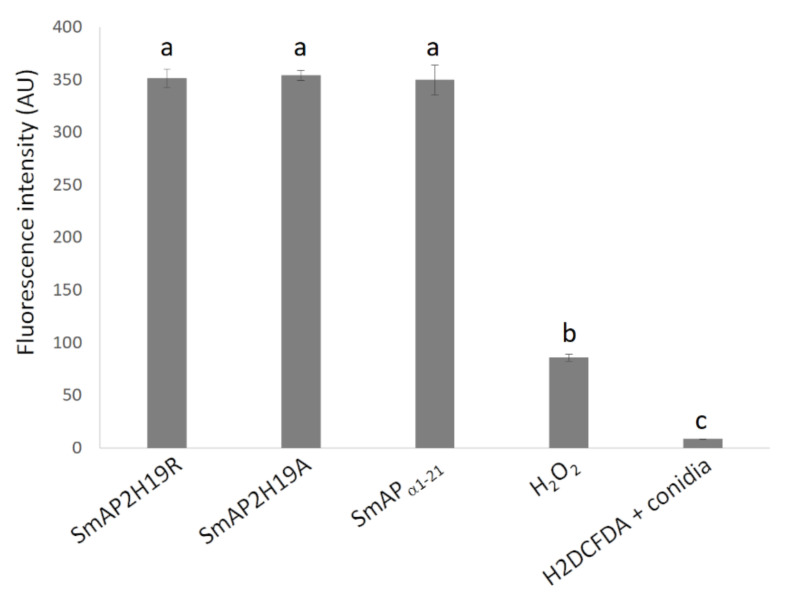
Quantitative fluorescence of conidia treated with peptides SmAP_α1-21_, SmAP2H19R, and SmAP2H19A at micromolar concentrations and H_2_DCFDA (10 µM), H_2_O_2_ (88 mM), or water (H_2_DCFDA + conidia) for 90 min. Fluorescence intensity is expressed in arbitrary units. Bars are mean values ± SD from 3 replicates and 2 independent assays (n = 6). Treatments with same letter do not significantly differ (*p* > 0.05).

**Figure 7:**
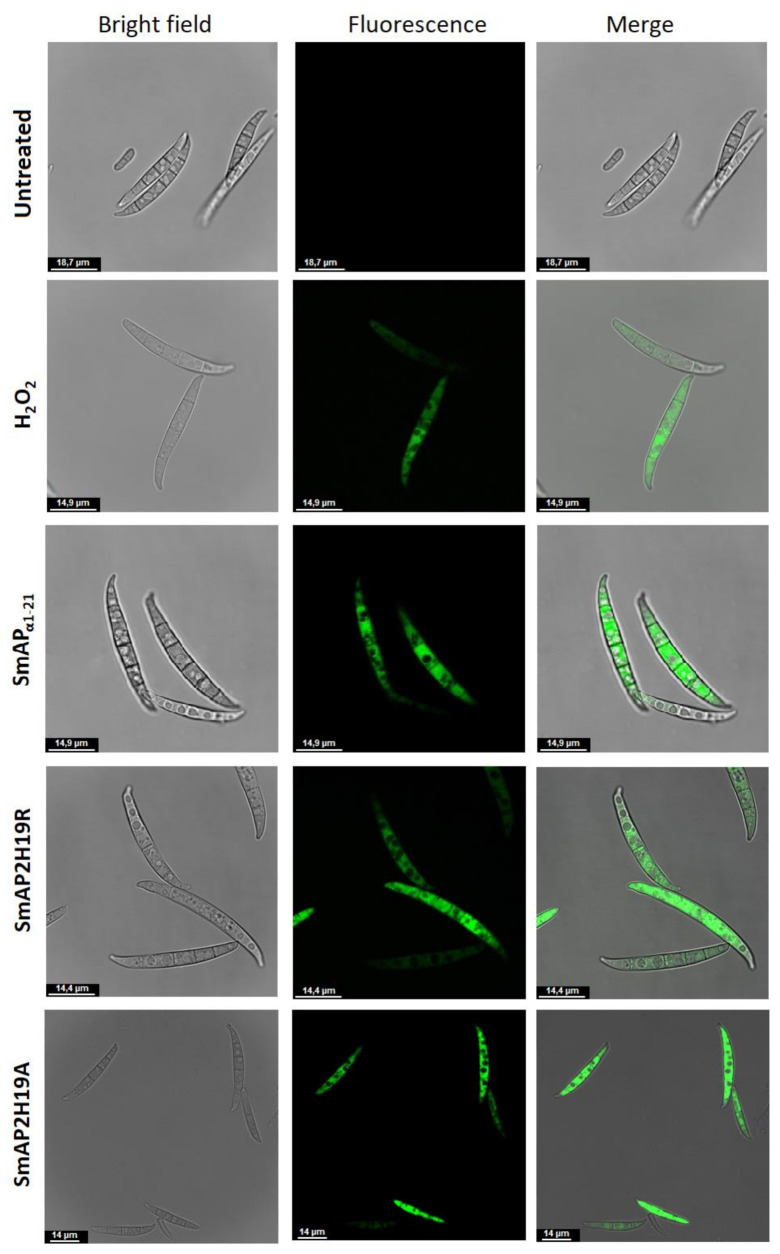
Representative confocal fluorescence images showing production of reactive oxygen species (ROS) by *Fusarium graminearum* in response to treatment with peptides SmAP_α1-21_, SmAP2H19R, and SmAP2H19A at their MIC for 1.5 h. Bright-field, fluorescence, and merged images are shown as different columns. H_2_DCFDA probe was used at 10 µM (λ_ex_ 492 nm and λ_em_ 527 nm). Bar indicates size in μm.

The 2’,7’-dichlorodihydrofluorescein diacetate (H_2_DCFDA) probe allows evaluating the in situ presence of different ROS. This probe can diffuse into the cell where it is deacetylated by cellular esterases to a nonfluorescent compound. In the presence of ROS, the nonfluorescent compound is oxidized to 2’,7’-dichlorofluorescein (DCF), a highly fluorescent species. The evaluation of ROS in conidia was carried out through fluorometry (Figure 6) and CLSM (Figure 7). After 30 min of incubation, fluorescence was not observed in the conidia treated with SmAP_α1-21_, SmAP2H19R, or SmAP2H19A with neither of these methodologies (data not shown). The production of ROS, however, was verified after 1.5 h of incubation with all the peptides assayed demonstrating that the induction of oxidative stress could be part of their antifungal mechanism and suggesting a particular temporal dynamic for this process. 

**Figure 8:**
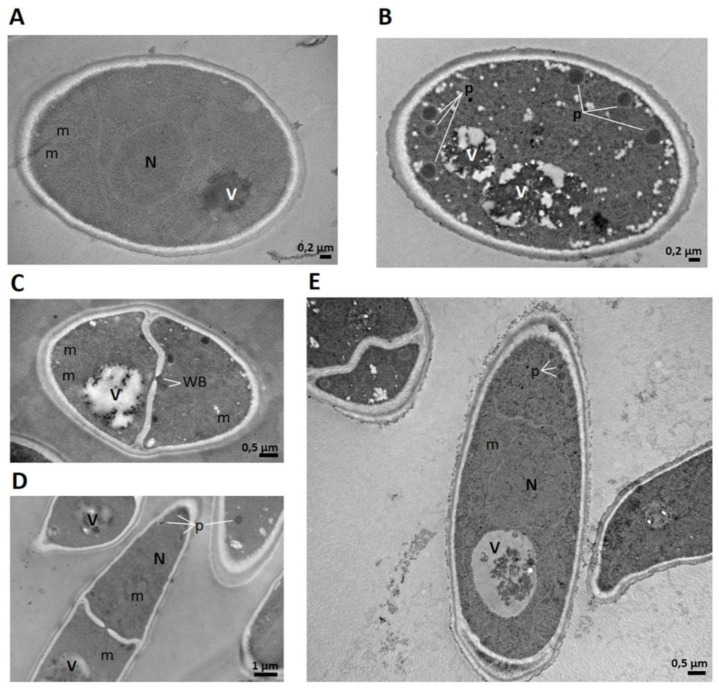
Transmission electron microscopy (TEM) images of *Fusarium graminearum* conidia incubated with peptide SmAP_α1-21_. (**A**) Untreated conidia cross-section, (**B**,**C**) cross-section; and (**D**,**E**) longitudinal section of treated conidia. In addition to morphological changes in the cell wall and cytoplasm, numerous electron-dense peroxisomes are observed near the cell membrane in treated cells, as well as Woronin bodies toward pore septa (p: peroxisomes; m: mitochondria; N: nucleus; V: vacuole, WB: Woronin body). Bar indicates size in μm.

We previously reported the presence of a greater number of peroxisomes in conidia treated with SmAP_α1-21_ compared with untreated cells [21]. In the present work, we confirmed this observation (Figure 8): an increase in electron density and the presence of many electron-dense peroxisomes around the membrane and cell wall were detected in the treated conidia. 

### 2.5. His19 Is Essential for SmAP_α1-21_ Internalization

**Figure 9:**
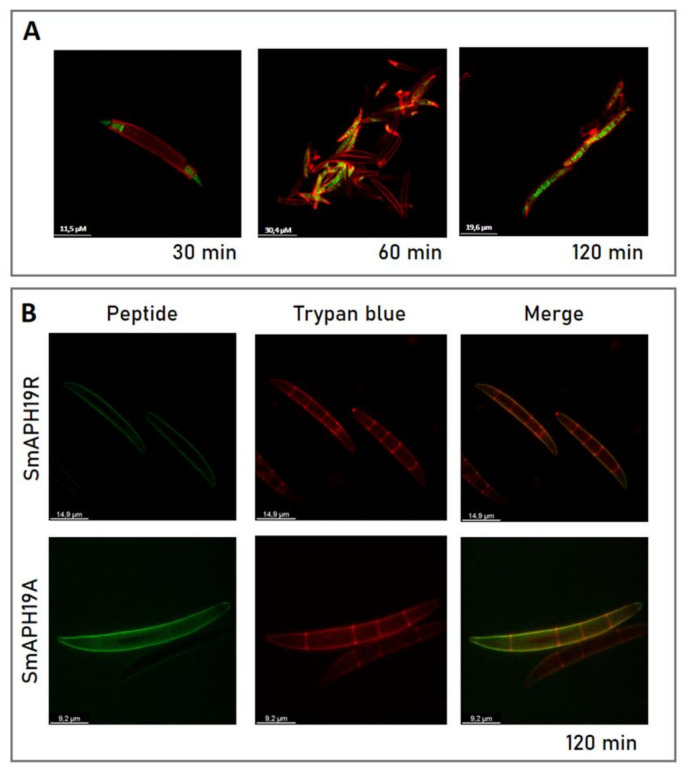
(**A**) Subcellular localization of SmAP_α1-21_ peptide in *Fusarium graminearum* conidia incubated at MIC for indicated periods of time. (**B**) SmAP2H19R and SmAP2H19A at their MIC for 2 h. Bright-field, fluorescence, and merged images are shown as different columns. Resulting images of superimposition of the green (peptide) and red (cell-wall marker: trypan blue 10 μg/mL, λ_ex_ 633 and λ_em_ 533–700 nm) channels are observed. Peptides were derivatized with fluorescein (λ_ex_ 488 nm and λ_em_ 506–566 nm). Bar indicates size in μm. Confocal fluorescence images are representative.

We studied the antifungal action of peptides by CLSM using fluorescently labeled peptides. To examine the correlation between antifungal activity and localization of SmAP2H19R and SmAP2H19A, as well as its parent peptide SmAP_α1-21_, we used fluorescein or rhodamine B (F- or RB-) labeled peptides to determine their localization in conidia after incubation. For these assays, trypan blue dye was used as a fungal cell-wall marker since it can emit an intense red fluorescence after binding to cell-wall chitin and glucans. Regardless of the label used, the same results were obtained for each peptide; for simplicity, images of fluorescein-labeled peptides are shown in Figure 9. First, conidial germination inhibitory activities of labeled peptides were tested.

For F-SmAP_α1-21_ and RB-SmAP_α1-21_, a slight loss in the inhibitory activity of conidial germination after treatment was observed when compared with the unlabeled peptides (Table 1), whereas the activity of the other two peptides remained unaffected. Labeled peptides F-SmAP2H19R and F-SmAP2H19A localized toward the extracellular region of the conidia, colocalizing with the cell-wall marker (Figure 9B). In the internal septa of the conidia, no green fluorescence was observed, suggesting that the peptides did not enter the cell at the assay conditions. On the contrary, at short incubation times (30 min), labeled F-SmAP_α1-21_ began to internalize in the conidia, first entering through the basal and apical cells of the macroconidia. As the incubation times were prolonged, the green fluorescence localized in all the cells of the spores, with a nonhomogeneous distribution in the cell cytoplasm (Figure 9A).

## 3. Discussion

In the search for novel highly active plant AMPs, we focused on wild species. Wild plants and weeds naturally exhibit resistance to pathogens due to their perfect adaptation to the environment, making them a valuable but unexplored source of natural AMPs [25]. Previously, our research group designed synthetic peptides from the α-core motif (SmAP_α1-21_, SmAP_α10-21_) of the putative defensin DefSm2-D from the wildflower *S. marianum*. SmAP_α1-21_ and SmAP_α10-21_ inhibited the growth of the phytopathogen *F. graminearum* at low micromolar concentrations and produced membrane permeabilization. Additionally, the peptide SmAP_α1-21_ induced morphological changes on the conidia cell wall and the cytoplasm. The fungal cell wall constitutes a promising target for the development of antifungal compounds due to its unique biochemical and structural organization, which is absent in plant and mammalian cells [21]. 

In the present work, we proposed to study the relevance of His19 in the peptide SmAP_α1-21_, and two new peptides were designed for that purpose. The modified peptides, SmAP2H19R and SmAP2H19A, exhibited antifungal activity against *F. graminearum* at micromolar concentrations. It is common to find two or sometimes three consecutive arginines in several plant defensin γ-cores, usually next to one cysteine, as exemplified by the sequence RGFRRRC present in the MtDef4 γ-core [15]. The replacement of His19 with Arg in the peptide SmAP2H19R increased the net positive charge by 0.1 unit at pH 5.5 with respect to the parent peptide. This change, however, did not produce a significant effect on the antifungal activity, as the MIC and time-to-kill values remained close to those of SmAP_α1-21_. On the contrary, the replacement of His19 by Ala in the peptide SmAP2H19A produced a decrease in the net charge of one unit at pH 5.5 with a significant increase both in the MIC and time-to-kill values, thus evidencing the importance of the positive charge in some vicinity of position 19 of the antifungal peptide. The role of positive charges in AMPs is related to the electrostatic interactions that basic amino acids can establish with their targets at the target sites. Recently, Toledo et al. (2021) showed that increasing the positive net charge of a γ-core-derived peptide from VuDef1 (a *Vigna unguiculata* cowpea seed defensin) by changing original DD with RR residues improved its antifungal activity. In particular, the contribution of Arg residues to the activity of antimicrobial peptides has been well-described [26,27]. Arginine has a relatively long aliphatic side chain with a positively charged terminal guanidino group. When this residue is exposed to an interface, the aliphatic group is oriented toward the hydrophobic portion of the membrane; meanwhile, the guanidinium group is able to interact in the polar region with the negatively charged moieties of the phospholipids, a behavior known as snorkeling [22]. Although it is present in many AMPs, the role of His, as well as its contribution to the mechanisms of action, has not been studied in-depth, as happened with other residues, such as arginine or lysine [28]. Histidine can be involved in different types of interactions, and the type of interaction that prevails will fundamentally depend on the pH of the medium and the His environment in the peptide [29]. The physicochemical features of this residue allow it to establish interactions of diverse nature: the formation of coordination complexes with metal cations, cation–π, hydrogen–π, or π–π stacking interactions [30]. It has been shown that the imidazole group of a protonated histidine has a propensity to form similar charged contact pairs with other protonated histidine or with arginine, an interaction with similar strength to that of the arginine–arginine pairing, which is a relevant feature of cell-penetrating peptides [30,31].

Many plant defensins have been reported to bind to specific components of fungal cell membranes as an initial step in their mechanism of action; several defensins target different membrane lipids, although many of these lipids are located in the inner monolayer [13,14,32,33]. To study the peptides’ ability to insert into the lipid membrane, we used the monolayer technique. Langmuir monolayers mimic cell membrane surfaces, and they allow the study of the insertion phenomenon isolated from other changes in membrane lipid architecture [34]. A fungal membrane-like composition was used to build lipid monolayers at the air–buffer interface. Phosphatidylcholine is a common phospholipid in the plasma membranes of fungi and other eukaryotic organisms, whereas ergosterol is an integral part of the fungal cell membranes [16,35]. Using different techniques, other authors have shown the influence of ergosterol on the interaction of plant defensins in vitro [16,36]. Our results demonstrate that SmAP_α1-21_, SmAP2H19R, and SmAP2H19A interact with POPC–ergosterol monolayers after being injected into the subphase bulk, producing significant changes in the monolayer surface pressure. Moreover, the calculated MIP values were greater than 30 mN/m for all the peptides in this system. This value is considered to be equivalent to the lateral pressure in cell membranes [23,37], indicating that these peptides would have the capacity to penetrate fungal membranes. 

Through PI uptake, we showed that SmAP2H19R and SmAP2H19A produced the permeabilization of the plasma membrane of *F. graminearum* conidia after 1 and 1.5 h of peptide incubation, respectively. PI penetrates cells with damaged membranes and binds to nucleic acids. These results suggest that membrane permeabilization either contributes to the antifungal activity of SmAP2H19R and SmAP2H19A or is a consequence of the peptides’ action. Additionally, these results show that though the positive charge at position 19, included within the α-core region, is relevant for membrane permeabilization, it is not the sole determinant. Moreover, peptides derived from DefSm2-D α-core with His19 (SmAP_α1-21_ and SmAP_α10-21_) produced reversible conidia aggregation [21]. However, the replacement of His with Arg or Ala canceled this aggregation. A similar effect of cell aggregation was observed on the bacteria *Xanthomonas campestris* treated with MtDef5B, one of the two defensin domains from *M. truncatula* [38]. The simultaneous replacement of His-Arg with Ala-Ala in the two γ-core motifs of MtDef5 (His36 and Arg37 in domain A, and His93 and Arg94 in domain B) prevents the build-up of aggregates. The aggregation of conidia could be explained by interactions between components of the cell wall naturally exposed in conidia or revealed due to the stress produced by the peptides SmAP_α1-21_ and SmAP_α10-21_. Conidia of various filamentous fungi are coated with hydrophobins and melanins; it is known that when conidia are grown in submerged culture, conidial aggregation occurs via hydrophobic and electrostatic interactions of hydrophobins and melanins, respectively [39]. Electrostatic interactions result from van der Waals forces and negative charge repulsion from carboxyl groups in the conidial wall structure. Electrostatic interactions are also affected by counter-ions (cations) and the physiologic conditions of conidia that modify the carboxyl groups [40]. When the dormancy of conidia is broken and the conidia are swollen, the melanin and hydrophobin layer is broken, and the cell-wall polysaccharides become exposed on the surface [39]. Specific interactions between conidia wall components also occur when polysaccharides are exposed to liquid and contribute to conidia aggregation through salt bridging in submerged culture [40]. We proposed that designed peptides containing His19 may favor electrostatic interactions with negatively charged melanins or with exposed polysaccharides to produce aggregation in the stressed conidia.

In this work, the presence of a greater number of peroxisomes in the cell cytoplasm was verified when conidia were challenged with peptide SmAP_α1-21_ compared with untreated conidia. Peroxisomes are dynamic organelles that can be rapidly formed by the fungal cell depending on environmental conditions and the stage of development. De novo biogenesis and fission from existing peroxisomes are processes that the cell uses to increase the number of these organelles [41]. In filamentous fungi, peroxisomes are involved in various cellular metabolic processes. They participate in fatty acid β-oxidation intended for energy generation and/or in the production of cell-wall precursors (chitin and glucans), in the glyoxylate cycle, in the biosynthesis of secondary metabolites, and, together with the mitochondria, in the production and detoxification of ROS [42,43,44]. Likewise, in filamentous fungi, peroxisomes can form Woronin bodies (WBs); these protein-rich organelles are located adjacent to septa in mycelia, germ tubes, infective hyphae, and, less frequently, conidia. One of the functions described for WBs is pore sealing of hyphal septa after damage or injury, thus preventing cytoplasmic leakage [45]. The presence of peroxisomes in the conidia treated with SmAP_α1-21_ may account for the processes that took place because of peptide action, whether associated with ROS detoxification, the need for raw material to obtain energy or carbon structures for cell-wall remodeling, or through the formation of WB to seal septa and avoid damage expansion to all the cells within the conidium.

Whether permeabilization by SmAP_α1-21_ causes cell death by inducing outflow of cytoplasmic contents or by facilitating the entry of the peptide to access intracellular targets is still unknown. In this study, the ability of F-SmAP_α1-21_, F-SmAP2H19R, and F-SmAP2H19A to enter conidia cells was monitored by fluorescently labeled peptides and by using trypan blue dye as a cell-wall marker. F-SmAP_α1-21_ was able to enter and accumulate at high concentrations inside basal and apical cells during a short incubation period; the same was observed for RB-SmAP_α1-21_ (data not shown). Internalization was completed before 2 h of incubation. Interestingly, internalization was not observed for peptides RB-SmAP2H19R and RB-SmAP2H19A since these peptides remained in the extracellular region of the conidia even after 2 h of incubation. These results suggest that modified peptides F-SmAP2H19R and F-SmAP2H19A exert their antifungal action from the extracellular region of the fungal cell and additionally suggest that His19 has a crucial role in the internalization of the parent peptide F-SmAP_α1-21_.

The induction of ROS is hypothesized to be another significant event in the antifungal action of various plant AMPs, including defensins [46,47]. For example, defensin RsAFP2 from *Raphanus sativus* mechanism of action on *Candida albicans* involves the production of ROS as a consequence of a signaling cascade induced after defensin binding to glucosylceramide [48]. Excessive production of ROS and its accumulation in the cell cytoplasm can lead to oxidative stress, producing DNA, RNA, and mitochondrial damage, and lipid and protein oxidation, which can ultimately trigger programmed cell death [26]. Challenge of conidia with SmAP_α1-21_, SmAP2H19R, and SmAP2H19A induced ROS production. In the case of conidia exposed to peptides SmAP_α1-21_ or SmAP2H19R, ROS were detected only after 90 min, suggesting that this production occurs after cell membrane permeabilization and possibly because of it. Additionally, considering that the time-to-kill value of SmAP_α1-21_ and SmAP2H19R was between 1 and 3 h, ROS are likely contributing to the killing of conidia by these peptides. Concerning peptide SmAP2H19A, membrane permeabilization and ROS production occurred slower than with the parent peptide SmAP_α1-21_, which correlates with the longer time-to-kill value of SmAP2H19A. How SmAP_α1-21_, SmAP2H19R, and SmAP2H19A induce ROS production remains to be elucidated. Defensin NaD1 from *Nicotiana alata* induces ROS production in *F. oxysporum* hyphae. Interestingly, in this case, ROS was not detected at concentrations below those required to cause growth inhibition, even when membrane permeabilization was observed for NaD1. As a result, Van Der Weerden et al. (2008) [49] concluded that membrane permeabilization, whereas required, may not be sufficient to cause the cell death of *F. oxysporum* hyphae treated with NaD1. In the same vein, the sequestration of ROS in yeast treated with RsAFP2 can inhibit its antifungal activity [48].

We conclude that SmAP_α1-21_ has a multistep mechanism of action against *F. graminearum* conidia; this mechanism involves fungal cell-wall structural alteration, peroxisome biogenesis, membrane permeabilization, and induction of oxidative stress; the peptide also has the potential to interact with plasma membranes. The change of His19 for Ala or Arg did not suppress membrane permeabilization, ROS production, and cell death by peptides derived from the DefSm2-D α-core. The replacement by Ala, however, turned all the processes slower. The extracellular localization of peptides highlights the role of the His19 residue in the internalization event. We propose that SmAP_α1-21_ internalization can occur in two different ways, i.e., as in the case of MtDef5 and HsAFP1 defensins through the endocytic pathway or as was shown for NaD1 defensin through peptide membrane translocation [50,51].

## 4. Materials and Methods

### 4.1. Biological Material

*Fusarium graminearum* SP1 was isolated from a grain sample obtained from San Pedro, Buenos Aires, Argentina. The strain was previously characterized as highly pathogenic and toxigenic both in vitro and in vivo [52,53].

### 4.2. Peptide Design and Synthesis

Peptides were designed based on the synthetic peptide SmAP_α1-21_ (KLCEKPSKTWFGNCGNPRHCG), derived from the α-core motif of DefSm2-D [21]. We considered evaluating the importance of the His19 residue, and to that purpose, we generated two new peptides derived from SmAP_α1-21_: one altering its net charge by replacing His19 by Ala (peptide SmAP2H19A) and the other changing the His with the cationic residue Arg (peptide SmAP2H19R). 

Peptide sequences (Table 1) were synthesized using a Liberty Blue™ automated microwave peptide synthesizer (CEM Corp., Matthews, NC, USA) following a standard 9-fluorenylmethoxycarbonyl (Fmoc)/*tert*-butoxycarbonyl (tBu) protocol [54]. A Rink Amide resin (loading 0.74 mmol/g) was used as the solid support. Standard couplings of amino acids were carried out in *N,N*-dimethylformamide (DMF) using *N,N*-diisopropylcarbodiimide (DIC)/OxymaPure® activation and the corresponding amino acid. Fmoc removal was carried out with 20% *v*/*v* 4-methylpiperidine (4MP) in DMF. Deprotection and coupling were performed following a microwave method. After completion, peptides were cleaved from the resin in trifluoroacetic acid (TFA) with gentle shaking for 3 hours at room temperature in the presence of scavengers (TFA/triisopropylsilane (TIS)/2,2′-(ethylenedioxy) diethanethiol (DOT)/Water 95:2.5:2.5:2.5) to avoid oxidation. After filtration, the crude peptides were precipitated by adding cold diethyl ether, centrifuged, washed with cold ethanol five times, dried, dissolved in ultrapure water, frozen, and lyophilized. 

Only enantiomerically pure L-amino acids (Iris Biotech GmbH, Marktredwitz, Germany) were used throughout. Solvents for synthesis, deprotection reagents, and cleavage reagents were of synthesis grade and purchased from Merck KGaA (Darmstadt, Germany).

### 4.3. Peptide Purification and Characterization

Crude peptides were fractionated using preparative Clean-Up® CEC18153 extraction columns (UCT, Bristol, PA, USA) by washing the column twice with methanol and twice with water, loading 10 mg of the peptide dissolved in water onto the column, and eluting successive fractions with 10%, 15%, 20%, 25%, 30%, and 60% (*v*/*v*) acetonitrile–water. The fractions were evaporated using a Savant SPD 1010 SpeedVac Concentrator (Thermo Fisher Scientific, Asheville, NC, USA) and evaluated by reverse-phase–high-performance liquid chromatography (RP-HPLC) and electrospray ionization mass spectrometry (ESI-MS) to determine the main fraction containing the expected peptide.

Peptides were purified by RP-HPLC (Jasco Corporation, Tokyo, Japan) using a XBridge ™ BEH C18 column (Waters Corporation, Milford, MA) with a mixture of (A) H_2_O with 0.05% (*v*/*v*) TFA and (B) acetonitrile (ACN) containing 0.05% (*v*/*v*) TFA as mobile phase. For the elution of peptides, the gradient program was 8 min with 0–70% of B at 1 mL/min and detection at 220 nm. The molar mass of purified peptides was determined by HPLC-ESI-MS in an LCMS-2020 ESI-MS (Shimadzu Corp., Kyoto, Japan) in a 0–100% acetonitrile gradient for 20 min.

Theoretical physicochemical properties were calculated to complement the experimental results. To achieve this, Protparam (https://web.expasy.org/protparam/, accessed on 10 May 2022) and Protscale (https://web.expasy.org/protscale/, accessed on 10 May 2022) through the Expasy server and the peptide calculator through Bachem page were used (https://www.bachem.com/knowledge-center/peptide-calculator/, accessed on 12 May 2022).

### 4.4. In vitro Antifungal Assays

The peptides were tested for antifungal activity toward the filamentous fungus *F. graminearum* by performing hyphal growth inhibition assays according to the method of Bleackley et al. (2017) [55] with modifications introduced by Fernández et al. (2021) [21]. Aliquots (90 μL) of a 5 × 10^4^ spores/mL suspension were incubated for 48 h at 25 °C in a 96-well microplate with filter-sterilized peptide solutions (10 μL) at different concentrations in water. Germination of spores was evaluated by measuring the optical density at 595 nm using a microplate reader Infinite M200 Pro (Tecan, Männedorf, Switzerland) after 0, 19, 24, 43, and 48 h of incubation. Each test was performed in triplicate. The minimum inhibitory concentration (MIC) was determined as the minimum peptide concentration that completely inhibited fungal growth. Inhibition data were analyzed by one-way ANOVA, and the mean differences were evaluated at *p* < 0.05 using the Tukey test. Statistical analyses were performed using InfoStat software [56].

To determine the time-to-kill value of the synthetic peptides, half-strength potato dextrose broth (PDB) (5 mL) was inoculated with macroconidia from *F. graminearum* to a final concentration of 10^4^ conidia/mL. Peptides were added at their MIC, and the inoculated PDB was incubated for different periods: 0.5, 1, 3, and 6 h. A growth control was performed by incubating the conidia with water instead of peptide at 25 °C in the dark for 48 h. After each period, 100 μL of the 5 mL culture was added to 900 μL of sterilized water. This dilution was vortexed for 10 s, and 100 μL was plated onto three different half-strength PDA plates and incubated for 3 days at 25 °C in the dark before the colonies were counted. Peptide SmAP_α1-21_ was tested as a control in both in vitro assays. Each treatment was replicated twice.

### 4.5. Surface Pressure Measurement

Surface pressure experiments were carried out with a NIMA Langmuir trough Model 102M (NIMA Technology, Coventry, UK) with a Wilhelmy platinum plate as the surface pressure (π) sensor. The aqueous phase, or subphase, consisted of 20 mM Hepes, containing 150 mM NaCl, pH 7.4. The mixture of lipids, consisting of POPC–ergosterol (3:1) (Avanti Polar Lipids Inc. Alabaster, AL, USA) and dissolved in chloroform, was gently spread over the subphase surface until the desired initial surface pressure (π_o_) was attained (5, 10, 20, 30, and 40 mN/m). Monolayers were left for 15 min to allow complete solvent evaporation and film stabilization. SmAP_α1-21_, SmAP2H19R, or SmAP2H19A was injected with a micropipette into the subphase bulk (final concentration 20 µM), and the increment in surface pressure was recorded until a stable signal was obtained (π_eq_). At this equilibrium state, the total increment in surface pressure (Δπ_eq_ = π_eq_ − π_o_) produced by the peptides was determined. The plots of Δπ_eq_ as a function of π_o_ for each peptide allowed the determination of their maximum insertion pressures (MIPs) from the intersection of the linear regression curves with the x-axis (Δπ = 0).

The insertion curves (Δπ vs. t) were fitted according to Equation (1), and the time needed to reach half of Δπ_eq_ (τ) was obtained for different π_o_ of the lipid films [57].

Δπ= Δπ_eq_ t/(τ + t), (1)

All experiments were repeated at least three times for each peptide to ensure consistent results, and the data were analyzed using SigmaPlot (Systat Software, San Jose, CA, USA). Measurements were performed at 23 ± 1 °C.

### 4.6. Evaluation of Membrane Integrity

The effect of peptides at their MIC on *F. graminearum* conidia membrane permeability was assessed by visualizing the influx of the membrane impermeant fluorescent red dye propidium iodide (PI; Thermo Fisher Scientific) as described by Fernández et al. (2021) [21]. Spore suspensions (25 μL; ≈10^7^ spores/mL in water) were challenged with the peptide solutions prepared in water at their MIC and incubated for 30 min at 25 °C before visualization by fluorescence microscopy. An aliquot (5 μL) of PI (0.1 mM) was added to each suspension. After 30 min of incubation at 25 °C, the uptake of the fluorescence probe was evaluated at λ_ex_ 543 nm and λ_em_ 580 nm using a CLSM Leica TCS SP5 (Leica Microsystems GmbH, Wetzlar Germany). Images were processed with Leica Confocal Software (LCS) Lite v. 2.61.15. Spores that fluoresced red after incubation with PI were classified as damaged, whereas those unstained were classified as intact. Water and the commercial cationic surfactant cetyltrimethylammonium bromide (CTAB, 0.8 mM; Cicarelli, Santa Fe, Argentina) were used as negative and positive controls, respectively. Peptide SmAP_α1-21_ was tested as a control, but the results are not shown since they have already been published in a previous work [21]. Each experiment, consisting of two replicates per treatment, was performed twice.

### 4.7. Reactive Oxygen Species (ROS) Detection

Reactive oxygen species production triggered by each peptide was monitored by fluorometry using an Infinite M200 Pro microplate reader (Tecan, Männedorf, Switzerland) and CLSM with a Leica TCS SP5 microscope. In both cases, water and H_2_O_2_ were used as negative and positive controls, respectively. SmAP_α1-21_, SmAP2H19R, and SmAP2H19A were tested for ROS production.

For fluorometry assays, conidia suspensions (10^6^ spores/mL) were incubated in water with the peptides at their MIC for 30 min or 1 h at 25 °C, centrifuged for 10 min at 2250× *g*, and resuspended in phosphate-buffered saline (PBS, 25 mM pH 7.4). Then, H_2_DCF-DA probe was added at a final concentration of 10 µM, incubated for 30 min, and centrifuged again at the same conditions. The resulting pellet was resuspended in PBS. H_2_DCF-DA is deacetylated by cellular esterases to a nonfluorescent product (H_2_DCF). In the presence of ROS, this product is oxidized within the cells to form DCF that has a strong fluorescence. Assays were carried out twice with three replicates per sample (n = 6). ROS detection data were analyzed by one-way ANOVA, and the mean differences were evaluated at *p* < 0.05 using the Tukey test. Statistical analyses were performed using InfoStat software [56]

For CLSM, conidia suspensions (10^7^ spores/mL) were incubated in water with the peptides at their MIC for 1 h at 25 °C; the probe was added at a final concentration of 10 µM and incubated for 30 min. The ROS probe was excited at 488 nm with argon ion laser, with the emission window set at 510 to 560 nm. Images were analyzed using LCS Lite v. 2.61.15. Sequential bright-field images were captured with a transmitted light detector. CLSM studies were carried out in at least two independent assays and visualized in three independent samples each time (n = 6).

### 4.8. TEM Imaging

TEM was used to study the abundance of peroxisomes in *F. graminearum* conidia (2 × 10^7^ spores/mL) exposed to 32 µM SmAP_α1-21_ for 1 h at 25 °C and then prepared for electron microscopy imaging. The sample was processed according to Fernández et al. (2021). A negative control was performed with water. Images were taken with a JEM 1200 EXII (Jeol Ltd., Akishima, Tokyo, Japan) microscope located at the TEM Service, Facultad de Ciencias Veterinarias, UNLP. Images were analyzed with ImageJ software (NIH, Bethesda, MD, USA, version 1.53q).

### 4.9. Peptide Derivatization and Subcellular Localization of Derivatized Peptides

Derivatized peptides were synthetized following the Fmoc/tBu protocol. Once peptide synthesis was completed and prior to the cleavage and deprotection of side groups, the peptides SmAP_α1-21_, SmAP2H19A, and SmAP2H19R were derivatized with fluorescein (5(6)-carboxyfluorescein, Novobiocehm) and rhodamine B (Sigma). After the deprotection of the last amino acid coupled to the peptidyl resin, the corresponding probe was incorporated. The coupling was carried out in two stages: the first using 3 excesses of HBTU (2-(1H-benzotriazol-1-yl)-1,1,3,3-tetramethyluronium hexafluorophosphate) in the presence of OxymaPure® and DIEA (N, N’-diisopropylethylamine) for 3 h at 135 rpm and 25° C, and the second with 3 excesses of TBTU (2-(1H-benzotriazol-1-yl)-1,1,3,3-tetramethyluronium tetrafluoroborate), in the presence of OxymaPure® and DIEA for 4 days under the same conditions. Coupling probes were used in a threefold excess, and all reagents were dissolved in a small DMF volume. To check the coupling end, a solution of bromophenol blue at 0.5% in DMF was used; if the resin turned blue, the second coupling was made, changing the activator for HBTU. After the second coupling, the resin was rechecked with bromophenol blue. 

Once the probe was incorporated, the side chains were deprotected, and the peptide cleaved from the polymeric support with TFA/TIS/Dot/ultrapure H_2_O (92.5/2.5/2.5/2.5). After filtration, the crude labeled peptides were precipitated by adding cold diethyl ether, centrifuged, washed with cold ethanol five times, dried, dissolved in ultrapure water, frozen, and lyophilized.

Then, labeled peptides were purified in C18 columns (Clean-up® CEC18123, UCT) using different percentages of acetonitrile (10–100%). As confirmed by RP-HPLC (XBridge™ BEH C18 column with a gradient of acetonitrile in 0.05% TFA/water at 1 mL/min flow rate), labeled peptides were obtained at 30% acetonitrile, whereas a minor fraction of unlabeled peptide eluted at 10% acetonitrile.

CLSM was performed to monitor possible internalization and subcellular localizations of fluorescently labeled peptides in *F. graminearum* conidia. Macroconidia (50 µL of 10^7^ spores/mL) were treated with fluorescein-peptides at their MIC, incubated for 2 h at 25 °C in the dark, and labeled with the cell-wall selective dye trypan blue (final concentration: 10 μg/mL) before mounting on a microscope for imaging. The TCS SP5 microscope was used for confocal imaging at room temperature in a dark room. Fluorescein-peptides were excited at 488 nm with an argon ion laser, and fluorescence was detected at 506–566 nm. Trypan blue was excited at 633 nm with a Helio-Neon laser and detected at 533–700 nm. Bright-field images were taken with a transmitted light detector. The laser intensity and laser exposure of the cells were kept to a minimum to reduce photobleaching and fungal cell damage. Images were analyzed using LCS Lite v. 2.61.15.

## Figures and Tables

**Table 1:** Main properties of SmAP_α1-21_ derived peptides.

Peptide	Sequence	Molecular Weight (Da)	pI ^1^	Net Charge ^2^	MIC (μM) ^3^
SmAP_α1-21_	KLCEKPSKTWFGNCGNPRHCG	2361.7	9.1	+4.0	32
SmAP2H19R	KLCEKPSKTWFGNCGNPR**R**CG	2383.8	9.5	+4.1	38
SmAP2H19A	KLCEKPSKTWFGNCGNPR**A**CG	2297.7	9.1	+3.0	100
F-SmAP_α1-21_ ^4^					60
F-SmAP2H19R					38
F-SmAP2H19A					100
RB- SmAP_α1-21_ ^5^					60
RB-SmAP2H19R					40
RB-SmAP2H19A					100

^1^ Theoretical pI was calculated using the ExPASy tool Compute pI/Mw (https://web.expasy.org/compute_pi/, accessed on 3 May 2022). ^2^ Net charge was calculated at pH 5.5 (pH of half-strength PDB in which the antifungal test was performed). ^3^ Minimum inhibitory concentration (MIC) is considered the minimal peptide concentration that completely inhibits *Fusarium graminearum* growth.^4,5^ F- and RB- refer to the fluorescein- and rhodamine-B-labeled peptides, respectively.

## Data Availability

Not applicable.
